# Specificity and stability of the *Acromyrmex*–*Pseudonocardia* symbiosis

**DOI:** 10.1111/mec.12380

**Published:** 2013-07-30

**Authors:** S B Andersen, L H Hansen, P Sapountzis, S J Sørensen, J J Boomsma

**Affiliations:** * Centre for Social Evolution, Department of Biology, University of Copenhagen2100, Copenhagen, Denmark; † Molecular Microbial Ecology Group, Department of Biology, University of Copenhagen2100, Copenhagen, Denmark

**Keywords:** 454 pyrosequencing, attine ant mutualism, bacterial community, *Pseudonocardia*

## Abstract

The stability of mutualistic interactions is likely to be affected by the genetic diversity of symbionts that compete for the same functional niche. Fungus-growing (attine) ants have multiple complex symbioses and thus provide ample opportunities to address questions of symbiont specificity and diversity. Among the partners are Actinobacteria of the genus *Pseudonocardia* that are maintained on the ant cuticle to produce antibiotics, primarily against a fungal parasite of the mutualistic gardens. The symbiosis has been assumed to be a hallmark of evolutionary stability, but this notion has been challenged by culturing and sequencing data indicating an unpredictably high diversity. We used 454 pyrosequencing of 16S rRNA to estimate the diversity of the cuticular bacterial community of the leaf-cutting ant *Acromyrmex echinatior* and other fungus-growing ants from Gamboa, Panama. Both field and laboratory samples of the same colonies were collected, the latter after colonies had been kept under laboratory conditions for up to 10 years. We show that bacterial communities are highly colony-specific and stable over time. The majority of colonies (25/26) had a single dominant *Pseudonocardia* strain, and only two strains were found in the Gamboa population across 17 years, confirming an earlier study. The microbial community on newly hatched ants consisted almost exclusively of a single strain of *Pseudonocardia* while other Actinobacteria were identified on older, foraging ants in varying but usually much lower abundances. These findings are consistent with recent theory predicting that mixtures of antibiotic-producing bacteria can remain mutualistic when dominated by a single vertically transmitted and resource-demanding strain.

## Introduction

Questions of conflict and cooperation are fundamental for understanding the evolutionary stability of genomes, societies and inter-specific mutualisms (Herre *et al*. 1999; Burt & Trivers 2006; Bourke 2011; Scheuring & Yu 2012; Schluter & Foster 2012). This is particularly apparent when considering multispecies symbioses consisting of a mixture of mutualistic and parasitic partners with potentially diverging fitness interests, such as microbial gut communities (Qin *et al*. 2010), nitrogen-fixing bacteria of legumes (Kiers *et al*. 2003) and microparasites of wild voles (Telfer *et al*. 2010). There is an ongoing debate on which mechanisms are primarily responsible for maintaining evolutionary stability of mutualistic interactions. Mutualisms that involve lifetime commitments between a single host and symbiont strain tend to have efficient competitive exclusion mechanisms to maintain symbionts in monoculture (Poulsen & Boomsma 2005; Aanen *et al*. 2009), so that symbionts can remain maximally cooperative (Frank 1996). However, many mutualisms have multiple strains competing for the same functional niche and continue to acquire new ones throughout life. Such dynamics may either require host screening of candidate symbionts (Archetti *et al*. 2011a,b; Scheuring & Yu 2012) or host sanctions against underperforming symbionts to maintain a high level of cooperation (Kiers *et al*. 2003; Kiers & Denison 2008). Obtaining accurate information on diversity of symbionts from individual hosts is therefore relevant for understanding the extent of cooperativeness in mutualisms.

The symbiosis between fungus-growing (attine) ants and their microbial symbiont community is an intriguing example of a complex mutualism involving multiple partners. The ants farm basidiomycete fungi for food in underground nest chambers and are completely dependent on this lifestyle, which evolved *c*. 50 million years ago (Schultz & Brady 2008). As far as this has been investigated, the cultivar is reared as a monoculture (Poulsen & Boomsma 2005; Mueller *et al*. 2010b), a practice that in leaf-cutting ants is reinforced by antagonistic behaviour of both the ants and the resident fungus towards unrelated strains (Bot *et al*. 2001; Poulsen & Boomsma 2005; Ivens *et al*. 2009). In contrast to these effective measures to prevent competition with related, but genetically different strains of symbionts, the fungus garden is relatively vulnerable to infections by the specialized parasitic ascomycete fungus *Escovopsis* (Currie *et al*. 1999a, 2003c; Gerardo *et al*. 2006). To meet these infection challenges, the ants employ a range of behavioural (Currie & Stuart 2001), chemical (Fernández-Marín *et al*. 2009) and biological (Currie *et al*. 1999b) control measures. The latter are often achieved by the use of antibiotic compounds from actinomycete bacteria, housed in specialized structures on the ant cuticle (Currie *et al*. 2006).

Similar to the symbiosis between the ants and their crop fungus, the association with cuticular actinomycetes has also been thought to represent an ancient co-evolved mutualism characterized by a single lineage of bacteria. The initial studies of diversity of the cuticular bacteria of attine ants were carried out by culturing and sequencing isolates, which revealed monocultures of what was ultimately identified as *Pseudonocardia* bacteria (Currie *et al*. 2003b; Cafaro & Currie 2005). It was thus inferred that each ant colony has a single particular strain (Cafaro & Currie 2005; Poulsen *et al*. 2005), that transmission is vertical with newly eclosed workers and virgin queens obtaining these bacteria from their sisters and/or the (likewise vertically transmitted) fungus garden (Poulsen *et al*. 2003a) and that varying degrees of co-evolution between the ant hosts and the bacteria occur (Cafaro & Currie 2005). However, controversy has arisen over the extent to which: 1. ants and bacteria are sufficiently faithful to each other to make co-evolution likely, 2. the actinobacterial antibiotics are specifically targeted towards, and coevolved with, both *Escovopsis* and the ants and 3. the growth form of bacterial cultures on the surface of ant workers is a multispecies biofilm rather than a monoculture (Mueller *et al*. 2008, 2010a; Sen *et al*. 2009; Barke *et al*. 2010; Cafaro *et al*. 2011; Ishak *et al*. 2011; Mattoso *et al*. 2011; Caldera & Currie 2012).

Part of the criticism focused on the limitations of using culturing methods to infer bacterial diversity and on the use of selective media that favour *Pseudonocardia* but may limit the growth of other bacteria (Mueller *et al*. 2008). Later, culturing studies have indeed found a greater diversity on attine ants with, among others, actinomycete genera such as *Streptomyces* and *Amycolatopsis* being present and sometimes abundantly so (Kost *et al*. 2007; Haeder *et al*. 2009; Sen *et al*. 2009; Barke *et al*. 2010; Schoenian *et al*. 2011). However, recent studies documented that specific *Pseudonocardia* lineages are consistently associated with genera of attine ants in spite of having close free-living relatives (Cafaro *et al*. 2011) and confirmed that antibiotics derived from cuticular *Pseudonocardia* protect *Acromyrmex* gardens against *Escovopsis* (Poulsen *et al*. 2010). Also, the finding of considerable population structure between ants, bacteria and parasites in the *Apterostigma dentigerum* system supports a role of co-evolution (Caldera & Currie 2012). A recent modelling study by Scheuring & Yu (2012) appears to reconcile these opposing views by showing that a dominant vertically transmitted ‘native’ mutualistic symbiont can stably co-exist with a series of environmentally acquired bacteria as long as competition between all strains is universally driven by antibiotic production aimed at harming competing strains. This model would be supported if cuticular diversity on newly hatched ants would appear to be a monoculture of vertically transmitted symbionts and increased diversity by acquisition of environmental bacteria restricted to later developmental host stages.

Natural diversity of bacterial communities can be estimated with high-throughput sequencing, a technique that identifies most if not all unculturable bacteria, although PCR bias may induce some noise in estimations of proportional abundance. The first surveys with 454 sequencing included three fungus-growing ant genera, *Trachymyrmex septentrionalis* (four ants from one laboratory nest), *Cyphomyrmex wheeleri* (four ants from one laboratory nest) and *Mycocepurus smithii* (four ants from two laboratory nests) and showed that these ants carried multiple *Pseudonocardia* species in addition to a wide range of other bacteria, several of which could also be cultured (Sen *et al*. 2009). However, as this sequencing was based on extracts of whole ants, this does not preclude that a number of these bacteria were associated with the soft tissues rather than the cuticular surface. Another potential bias is that ants that have been kept in the laboratory may have secondarily acquired bacteria that they would not associate with in the field, particularly if they would be older, foraging ants that have lives outside their colony.

In the present study, we revisit the questions of diversity, specificity and stability of ant cuticular bacterial communities (points 1 and 3 above), in light of the Scheuring & Yu (2012) hypothesis (see also Schluter & Foster 2012 for a more general model making similar predictions). We focus on the leaf-cutting ant *Acromyrmex echinatior*, which was not included in the study by Sen *et al*. (2009), but has been well studied for its phenotypic associations with actinomycete bacteria. After a first experimental study showing that cuticular *Pseudonocardia* respond to *Escovopsis* challenges (Currie *et al*. 2003a), further work identified that the cuticular growth patterns on newly hatched (callow) large workers are very predictable, with the laterocervical plates of the propleura (the ventral prothorax) being quickly colonized to produce a characteristic bacterial bloom and most ants becoming almost completely covered over the following 2 weeks (Poulsen *et al*. 2003b). After this peak, bacterial abundance gradually declines until only the laterocervical plates remain covered about a month later (Poulsen *et al*. 2003b). This location appears to be particularly adapted to harbouring bacterial cultures because it is speckled with cuticular tubercles, each supplied with unknown secretions of subcuticular glands (Currie *et al*. 2006).

While detailed morphological adaptations such as crypts and tubercles to feed actinomycetes are consistent with a long history of bacterial domestication and co-evolution with the ants (Currie *et al*. 2006), this does not necessarily imply that there has been strict co-cladogenesis or tight co-evolution between either the ants or the bacteria with specific lineages of *Escovopsis* (Mueller *et al*. 2008, 2010a). On one hand, the open ‘external’ location of the cuticular crypts should make it relatively easy for environmental bacteria to invade, which could lead to considerable symbiont diversity, as for example in the zooxanthellae of corals (Knowlton & Rohwer 2003) and rhizobial bacteria of legumes (Kiers & Denison 2008). On the other hand, the ants should remain under selection to make their glandular secretions so specific that they preferentially enhance the growth and competitive abilities of bacterial cultures that produce useful antibiotics, be it *Pseudonocardia* or other lineages (Boomsma & Aanen 2009; Barke *et al*. 2011; Scheuring & Yu 2012; Schluter & Foster 2012). To advance our understanding of the true nature, ontogeny and diversity of the bacterial cultures on the cuticle of *Acromyrmex* leaf-cutting ants, representing the crown group of the attine phylogeny, we set out to obtain a detailed culture-independent estimate of bacterial diversity on the laterocervical plates and adjacent parts of the pronotum of *A. echinatior*, and to compare these estimates across field and laboratory samples of the same colonies across a range of years.

## Materials and methods

### Ant sampling

**Figure 1:**
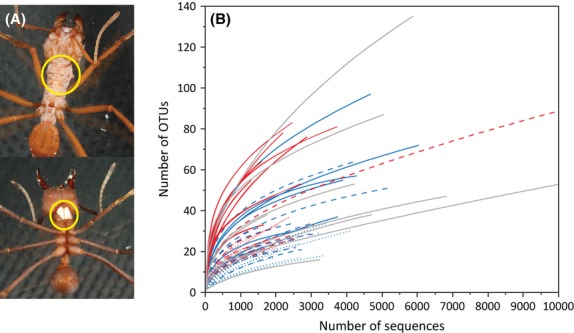
Bacterial abundance and diversity data obtained by 454 sequencing. (A) Dorsal view (top) of a callow worker of *Acromyrmex echinatior* covered in *Pseudonocardia* bacteria and ventral view (bottom) of a mature worker with bacterial growth concentrated on the laterocervical plates. Yellow circles indicate the parts of the cuticle that was dissected and sequenced. (B) Rarefaction curves of sequencing depth, each representing an individual sample and showing the observed number of OTUs as a function of simulated sequencing effort. Red curves represent samples from cluster 1 and blue curves samples from cluster 2 (see Fig. 2). Dotted curves are callow laboratory ants, dashed curves mature laboratory ants and solid curves field-collected ants. Curves based on other attine ant samples than *A. echinatior* and samples of *A. echinatior* that could not be placed in either cluster 1 or 2 are plotted in grey.

To assess cuticular bacterial diversity, large worker ants of two age categories were sampled from laboratory colonies of *Acromyrmex echinatior*. First, the laterocervical plates and pronotum of callow nurse workers were dissected. These ants were almost completely covered in bacteria as is typical for large workers of *A. echinatior c*. 2 weeks after eclosing (bacterial cover scales 10–12 according to the classification of Poulsen *et al*. (2003b); Fig. 1A). Dissection was performed in a Petri dish (sterilized with bleach) with tweezers (wiped with bleach, dipped in 96% ethanol and flame sterilized between samples) under a stereomicroscope. The attached soft tissue was carefully removed from the insides of dissected cuticular fragments to minimize the presence of *Wolbachia* endosymbiotic bacteria that are abundant in the thoracic muscles (Andersen *et al*. 2012). From the same colonies and two additional ones, we also sampled an older ant that only had visible bacterial growth on the laterocervical plates (Fig. 1A). These were ants with darker cuticles, representing cover scales 1–3 (Poulsen *et al*. 2003b), that is, the final stages of bacterial cover that are typical for foragers. The first set of samples will be referred to as category C (callow) samples (*n* = 17, one ant each from 17 different colonies) and the second set as category M (mature) samples (*n* = 19, one ant each from 19 different colonies; Table S1, Supporting information). The two categories were chosen to assess whether bacterial diversity changes with ant age, that is, whether bacterial diversity would be lower on callow workers that were confined to the fungus garden and entirely covered in bacteria at the time of sampling, compared to mature workers or vice versa. In addition, the collection of multiple samples from 17 colonies (and 19 colonies overall), allowed us to ask whether there are differences in bacterial community composition within and between colonies. All ants were sampled from within the fungus garden of colonies that had been collected in Gamboa, Panama between 2001 and 2011, and subsequently kept in four culture rooms in Copenhagen at *c*. 25 °C and 70% relative humidity, each containing multiple colonies of different attine ants. Throughout their laboratory ‘tenure’, colonies received the same locally collected bramble leaves, fruit fragments and dry rice.

Older field-collected samples of mature workers were available for 10 of the sampled laboratory colonies and another colony not sampled in the laboratory. These had been stored in 96% ethanol at −20 °C in tubes containing multiple workers from the same colony. One mature worker from each of these colonies was oven-dried and the laterocervical plates were dissected. Freezer samples from another six field colonies of *A. echinatior* (one large worker for each colony) that were analysed by Poulsen *et al*. (2005) were also included, to represent the two clusters of *Pseudonocardia* strains identified in that study by sequencing the EF-1α gene of *Pseudonocardia*. All of these field-collected samples are referred to as category F (field; Table S1, Supporting information). In addition, six laboratory samples representing four other species of attine ants from the same Gamboa study site, sharing their culture rooms with some of the sampled *Acromyrmex* colonies, were collected. These six samples comprised the laterocervical plates of *Trachymyrmex zeteki* workers (two samples, each with three individuals pooled to compensate for body masses being smaller than for large workers of *A. echinatior*), *Cyphomyrmex costatus* workers (two samples, each with five individuals pooled), *C. longiscapus* workers (one sample with three individuals pooled) and *Acromyrmex volcanus* (one large worker; see Table S1, Supporting information for details).

### DNA extraction

The dissected cuticular fragments were placed individually in sterile 2-mL screw lid tubes with 0.1-mm glass beads (MO BIO laboratories, Inc.), and DNA was extracted with a MasterPure DNA purification kit (Epicentre Technologies), which targets Gram-negative and Gram-positive bacteria with about equal efficiency (Rantakokko-Jalava & Jalava 2002). In short, 300 μL tissue lysis buffer was added and the bacterial membranes disrupted in a FastPrep machine for 45 s at 4.5 speed. Instead of the proteinase K supplied with the kit, three μL of proteinase K (Invitrogen) was added followed by >25 min incubation at 65 °C with frequent vortexing. The samples were cooled and precipitated according to the manufacturer’s instructions and the DNA eluted in 35 μL TE buffer.

### Amplification of 16S rRNA by PCR and tag-encoded FLX 454 pyrosequencing

Bacterial DNA was amplified with the general bacterial primers 341F/806R spanning the hypervariable regions V3 and V4 (Masoud *et al*. 2011). PCR was performed in a final volume of 20 μL with 4 μL 5× Phusion HF buffer, 0.4 μL 10 mm dNTP mixture, 0.2 μL Phusion Hot Start DNA Polymerase (2 units/μL, Finnzymes), 1 μL of each primer (10 μm), 1 μL 10× diluted template and water at the conditions: 98 °C for 30 s, followed by 35 cycles of 98 °C for 5 s, 56 °C for 20 s and 72 °C for 20 s, and a final extension at 72 °C for 5 min. The samples were moved directly to ice and run on a 1% agarose gel containing ethidium bromide for 50 min. The specific bands were cut and purified from the gel using the Montage DNA gel extraction kit (Millipore).

A and B adaptors for emPCR and pyrosequencing were added to each sample together with a sample-unique tag in an additional PCR. This procedure was performed as above except that the forward primer was replaced by 59 differently tagged forward primers, to be amplified over only 15 cycles of PCR. The first 36 of these samples were provided with an A adaptor, LinA_341F_1–36, and the reverse primer with a B adaptor LinB_806R, whereas the last 23 samples received a B adaptor, LinB_341F_58–80 and the reverse primer with an A adaptor LinA_806R (Masoud *et al*. 2011; Bendtsen *et al*. 2012). The PCR product was run on a gel and purified as described above, after which the DNA concentration was quantified using a Quant-iT dsDNA High-Sensitivity Assay Kit and a Qubit fluorometer (Invitrogen). Amplicons were mixed accordingly to ensure an equal representation of each sample, and two-one-region 454 sequencing runs were performed on a GS FLX Titanium PicoTiterPlate (70X75) using a GS FLX Titanium Sequencing Kit XLR70 according to the manufacturer’s instructions (Roche). The A- and B-tagged samples were prepared for two separate 454 sequencing runs, where the first included all of the laboratory samples of *A. echinatior* and the second all of the field samples and the other species of attine ants.

### Data analyses

The data of both runs (NCBI Sequence Read Archive Accession no. SRA050635) were analysed using the mothur pipeline (version 1.26.0, Schloss *et al*. 2009; www.mothur.org; standard settings from the Schloss SOP were used unless specified otherwise. The used scripts were deposited in Dryad). The flowgrams of the two runs were trimmed (max bp differences in primer = 2, max bp differences in barcode = 1, minflows = 360, maxflows = 720) and denoised separately, after which the remaining sequences were trimmed (minimum length = 200 bp, max. homopolymer = 8, bp differences in barcode = 1, bp differences in primer = 2, removal of forward primer) and sorted by sample ID. The resulting files were merged and the unique sequences identified and aligned to the silva reference alignment (version 102 from www.mothur.org). The alignment (deposited in Dryad) was screened (start position = 6428, minlength = 220), filtered (vertical = T, trump =.) and clustered. Chimera sequences were identified with chimera.uchime and removed. Next, sequences were classified with a slightly modified RDP taxonomy file (v.9): six *Wolbachia* sequences were added to the file (GenBank Accession nos AF491887, AF491885, AF491888, AF491886, GQ275124 and a *Wolbachia* sequence from the present study; ‘*Wolbachia*’, Appendix S1, Supporting information) because the RDP taxonomy file only contained a single *Wolbachia* sequence. Subsequently all sequences classified as unknown and Cyanobacteria were removed from the alignment, as they likely represent contaminants from chloroplast/plant material. Also Rickettsiales sequences were removed after we checked that they were all *Wolbachia* by NCBI BLAST searching of representative sequences and therefore must have come from soft tissue (Andersen *et al*. 2012). For the remaining data set, a genetic distance matrix was generated (cut-off = 0.15), after which sequences were clustered into OTUs and classified using default settings (furthest neighbour clustering with the criterion that all sequences comprising a specific OTU were at least 97% similar to each other). The 97% similarity cut-off is most commonly used as a compromise between allowing for some genetic variation within OTUs on one hand and minimizing the probability of unjustified splitting due to sequencing errors on the other. We also explored the robustness of this criterion by considering a 99% cut-off for the *Pseudonocardia* OTUs (see below), but as that did not produce convincing evidence of multiple OTUs nested within the ones already identified, we performed all analyses based on the OTUs identified with the 97% similarity cut-off. For each OTU, a representative sequence was subsequently obtained by selecting the sequence that had the smallest total distance to all other sequences within the OTU, only considering those OTUs that had a prevalence of >5% in at least one sample. After that, these representative OTU sequences were compared to known sequences in a NCBI GenBank BLAST search (the read distribution across OTUs for all samples is deposited in Dryad).

OTU classification rarefaction curves were generated by estimating the number of OTUs observed in each sample for every 100 sequences obtained, using the mean of 1000 iterations with resampling without replacement. For each of the samples, the relative contribution of each OTU was calculated as the number of sequences contributing to that OTU out of the total number of sequences for the sample. This value was used to calculate the mean prevalence ± SE for each OTU across all samples. To obtain the mean overall prevalence of groups of phylogenetically similar OTUs across samples (e.g. three Rhizobiales OTUs), the summed prevalence of the given OTUs was first calculated per sample, after which this value was used to estimate the mean prevalence ± SE for all samples together (Table S2, Supporting information).

We used two types of diversity estimates to further analyse the composition of cuticular bacterial communities of the ants: alpha diversity as an independent assessment of the diversity within each sample and beta diversity to sort the individual samples according to their degree of mutual similarity (Whittaker 1972). Beta diversity comparisons were obtained by using the UPGMA algorithm in mothur. Clustering was based on the distances between samples calculated as theta values, which is a measure of dissimilarity between samples based on the relative abundance of the OTUs in the compared communities (Yue & Clayton 2005). An unweighted UniFrac analysis in mothur of the resulting tree was also used to test whether samples classified as carrying either of the two dominant *Pseudonocardia* strains clustered together (see below). The obtained distance matrix was further used for principal coordinates analysis (PCoA), using the first three principal coordinates for visualization of the sample clustering in a 3D scatterplot in CurlyWhirly (available as free download at http://bioinf.hutton.ac.uk). The prevalence of OTUs across samples was visualized in a heatmap in R (R Core Team 2012).

Prior to estimating alpha diversity, data were normalized by random subsampling in mothur of 1023 sequences from each sample separately. This eliminated two samples from the diversity index analysis, which had fewer sequences (Ae.488C and Ae.488M), but given the relatively high variance in sequencing depth between samples, this cut-off was chosen as an optimal compromise, allowing inclusion of almost all samples while retaining a high sequencing depth throughout. We subsequently estimated α-diversity in mothur with the inverse Simpson diversity index ranging between 1 and the number of OTUs present (using the mean of 1000 iterations), and compared diversities between samples with a two-way anova and Tukey–Kramer HSD post hoc testing in JMP 9.0.2 for Mac OSX.

## Results

### Data quality and read distribution

In the first run with 36 samples, 214 007 sequences of 446 202 passed the quality controls and data filtering (48%). In this trimming step, 99% of the discarded sequences were removed because of short read length. In the denoising step, likely sequencing errors were identified and removed, grouping the reads into 25 086 unique sequences. The distribution of reads per sample was skewed, as one sample contributed 10% of the sequences (Sample Ae.480M, Fig. S1A, Supporting information). In the second run with 23 samples, 98 073 sequences of 389 715 passed the quality control (25%). Here, 85% were discarded because of short read length, allowing 19 985 unique sequences to be identified in the denoising step. The relatively low yield in the second run suggests that these samples contained a higher amount of primer-dimers and other short sequences in spite of similar gel extraction of PCR products. The files with the remaining sequences were merged and 35 063 unique sequences identified. These were aligned to the reference database, and the alignments were subsequently screened and filtered. This retained a total of 310 166 sequences. Of these, 11 811 were identified as unique, clustered and checked for chimeras, leaving 4762 unique sequences of 308 307 sequences in total (Fig. S1A, light grey bars; Table S1, Supporting information).

Following classification, sequences identified as Cyanobacteria/chloroplast (25 528 sequences, only sample Ae.480M), Rickettsiales (*Wolbachia*, 95 894 sequences, most samples) and sequences classified as ‘unknown’ (335 sequences) were removed to focus as much as possible on cuticular bacterial diversity (Fig. S1A, dark grey bars, Table S1, Supporting information). The average proportion of *Wolbachia* sequences removed was 50% ± 4% SE for the callow laboratory workers, 39% ± 4% SE for the mature laboratory workers and 11% ± 4% SE for the mature field workers. The significantly higher proportion of *Wolbachia* sequences removed from laboratory samples (one-way anova: *F*_2,52_ = 28.38, *P* < 0.0001, Tukey’s HSD: *P* < 0.0001) may reflect that it was slightly easier to avoid including soft tissue for ants that had been stored in ethanol for years, but is also consistent with our earlier finding that *Wolbachia* densities in the pronotum of large workers increase significantly when *Acromyrmex* colonies are moved from the field to permanent laboratory culture (Andersen *et al*. 2012). This effect was similar for ants harbouring either of the dominant *Pseudonocardia* strains (Ps1 and Ps2; see below; *t*_43.12_ = −0.72, *P* = 0.48) as expected when there is no direct interaction between cuticular and soft-tissue symbionts. Rarefaction curves showed that a deeper sequencing would likely have revealed more (rare) OTUs (Fig. 1B). However, the 27 OTUs that contributed more than 5% of the total sequences in at least one sample of the total of 993 OTUs (after removal of the sequences identified as *Wolbachia* and chloroplast) contributed on average 93% ± 4% SE of the total sequences per sample (calculated as the overall sequence prevalence mean among samples across the 27 OTUs in the individual samples; Table S2, Supporting information).

### Actinobacteria diversity

Two of the 27 most abundant OTUs were *Pseudonocardia,* and they dominated the bacterial communities of *A. echinatior* samples with a mean overall prevalence of 74% of the total sequences. One of these (Ps1, Appendix S1, Supporting information) was identical to various *Pseudonocardia* strains isolated primarily from *Apterostigma*, *Trachymyrmex* and *Acromyrmex* species placed in clade IV in the phylogeny of ant-associated *Pseudonocardia* (e.g. GenBank Accession no. EU283925; Cafaro *et al*. 2011). The second OTU (Ps2, Appendix S1, Supporting information) was identical to strains from clade VI isolated from primarily *Acromymex* and a few *Trachymyrmex* ants (e.g. GenBank Accession no. EU928983; Cafaro *et al*. 2011). With one exception, these two strains did not co-occur on individual ants or in single colonies (see below), and they were found in both sequencing runs and could thus not be artefacts of sequencing errors. The two *Pseudonocardia* OTUs were distinct using the 97% similarity criterion. Relaxing this threshold to 99% similarity retained Ps2 largely unchanged (Fig. S1B, Supporting information), but split Ps1 into five putative OTUs: four common and one rare OTU that was only found in one sample (Fig. S1C, Supporting information). However, the differences responsible for this splitting were all restricted to two homopolymer regions, suggesting that these were likely to be sequencing errors (Appendix S2 & S3, Supporting information). Only Ps1 was found to be homopolymeric in both regions, which likely explains that only the classification of this OTU differed between the 97% and 99% similarity cut-offs. To further validate the 97% similarity OTU classification, we amplified part of the 16S rRNA region from 12 samples (three callow laboratory ants, three mature laboratory ants and six field ants) following Morón *et al*. (1999). These samples were directly sequenced at Eurofins MWG Operon (Germany) and found to be identical to the representative Ps1 sequences that we had obtained within the homopolymer regions. The chromatograms were, however, ambiguous for four basepairs. This was not resolved by sequencing of cloned PCR product, but comparison of all 454 sequences for six samples covering most of the diversity (*n* = 9058 sequences) showed that only three (0.03%) had any deviations for these basepairs (Appendix S3, Supporting information see also legend for Fig. S1, Supporting information). We therefore concluded that it is most parsimonious to interpret Ps1 as a single OTU and to use the terms strain and OTU interchangeably when referring to Ps1 and Ps2 (see supplementary material for further details). It was further worth noting that the 99% cut-off failed to group any of the *A. volcanus* sample reads with the *A. echinatior* OTUs, suggesting that in this case, we might have identified a distinct *A. volcanus* strain that is closely related to the *A. echinatior* OTUs.

The two *Cyphomyrmex costatus* samples harboured *Pseudonocardia* at lower prevalences (10–63%; 1 and 2 strains, respectively) while none was found in the *C. longiscapus* sample. This was also the case for the two *Trachymyrmex* samples, which primarily harboured the single OTU 255 (99% and 35% prevalence, respectively; Appendix S1, Supporting information) with 98% sequence similarity to *Amycolatopsis* previously isolated from *T. septentrionalis* (GenBank Accession no. JN413646; Ishak *et al*. 2011). The same *Amycolatopsis* was also found in the *C. longiscapus* sample, but at a prevalence of only 3%. As discussed in more detail below, one of the *T. zeteki* samples contained 61% Rhizobiales bacteria, which are potential contaminants from the ant oesophagal tissues not properly removed during dissection of these small ants (Boomsma lab, unpublished data), suggesting that *Amycolatopsis* bacteria dominate on the cuticle. No *Pseudonocardia* OTUs were found at a prevalence of more than 5% in the *Trachymyrmex* samples. Sen *et al*. (2009) and Cafaro *et al*. (2011) found *Pseudonocardia* on what was classified as *T. zeteki* and *T*. cf. *zeteki*, respectively, of which one must be the same species as the one we have under that name.

Four OTUs (OTU 3, 36, 38 & 41, Appendix S1, Supporting information) had their closest matches (99%) to sequences from isolates of the genus *Streptomyces* (Actinomycetales, Streptomycetaceae), but three of these were also 99% similar to genus-level sequences of Micrococcineae and *Kineococcus*, suggesting that the sequenced region has limited power for more accurate identification (assuming species identifications for the GenBank submissions are in fact correct). The representative sequences of these four OTUs were 94–97% similar to each other and 95–99% similar to a *Streptomyces* previously isolated from *Acromyrmex* ants (GenBank Accession no. FJ490543; Haeder *et al*. 2009). Given that *Streptomyces* has repeatedly been found to be associated with attine ants (Kost *et al*. 2007; Haeder *et al*. 2009; Barke *et al*. 2010; Schoenian *et al*. 2011), it seems likely that at least some of these OTUs represent this genus. The four OTUs were found in 17 samples at a prevalence higher than 5%, 11 of which were field samples and all were mature workers. Two other Actinobacteria OTUs were also identified in some of the samples: one belonging to the Solirubrobacterales (OTU 43, Appendix S1, Supporting information; Ae.480F at 9%) and one belonging to the Nocardioidaceae (OTU 37, Appendix 1; not identified to genus level, five mature laboratory samples and seven field samples at 5–40%; Table S2, Supporting information).

### Other bacteria

Two strains were identified as unclassified Acidobacteria (OTU 72 and 893, Appendix S1, Supporting information), one as Alphaproteobacteria in the Sphingomonadaceae (OTU 23, Appendix S1, Supporting information) and one as belonging to the Firmicutes (OTU 126, Lachnospiraceae). Some of the samples contained *Pseudomonadales* (OTU 4, 27, 42 and 113, Appendix S1, Supporting information), but these were only identified in samples from the second 454 run (both field-collected ants and a *C. costatus* sample) and may represent contaminants from either the DNA extraction or a PCR step, or bacteria only facultatively associated with the ants. Sample Ae.342F was dominated by an Enterobacteriaceae OTU (OTU 521, Appendix S1, Supporting information; 76%), which may also be a contamination of unknown origin (work on a variety of environmental samples of *Pseudomonas* and *E. coli* takes place in the same laboratory). In addition to the removed *Wolbachia* OTUs, some of the other OTUs were also suspected to be of ant soft-tissue origin. These included three Rhizobiales (OTU 8, 65 and 182, Appendix S1, Supporting information) reaching respective frequencies of 61% and 77% in one of the *T. zeteki* samples and in the *C. longiscapu*s sample. These OTUs were also found at lower prevalences in four *A. echinatior* samples. One of these OTUs had a 98% sequence similarity to bacteria previously suggested to be gut symbionts of various ant species (e.g. GenBank Accession no. FJ477647; Russell *et al*. 2009), while the others produced closest matches (99–100%; e.g. GenBank Accession no. JX171806 and FR749843) to environmental samples. An Entomoplasmataceae OTU also resembled bacteria found in association with ant guts (OTU 6, Appendix S1, Supporting information; 95% similarity, for example GenBank Accession no. GQ275130; Russell *et al*. 2009). One of the two Bacteroidetes (OTU 39, the other being OTU 259, Appendix S1, Supporting information) was 99% similar to bacteria previously identified in *Acromyrmex* ants (98%, GenBank Accession no. AF91883; Van Borm *et al*. 2002). In addition, one OTU from the Burkholderiales (OTU 46, Appendix S1, Supporting information) and two from the Xanthomonadales (OTU 233 and 523, Appendix S1, Supporting information) were identified, orders which have also previously been found associated with ants (Russell *et al*. 2009; Table S2, Supporting information). All of these other bacteria were only identified in a minority of the samples and may thus once more result from contamination from, for example the oesophagus during dissection. However, as their origin was not confirmed, they were included in the analysis.

### Analyses of beta diversity

**Figure 2:**
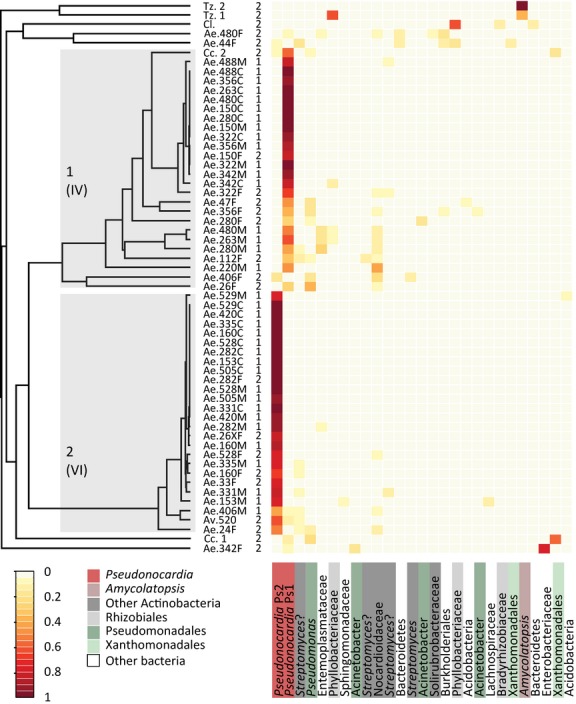
Hierarchical clustering of the bacterial communities from the cuticle of *Acromyrmex echinatior* and selected other attine ants, based on beta diversity analyses (Yue & Clayton 2005). On the *x*-axis, are the 27 most common OTUs, with the different taxonomic groups highlighted in different colours. On the y-axis, the dendrogram illustrates clustering of the samples and not phylogenetic relationships between the OTUs. Sample IDs are followed by the number of the sequencing run (1 or 2), and the frequency of each OTU is illustrated by a colour scale from light yellow (=zero to low frequency) to red (=high frequency to complete dominance). The two main clusters of *Acromyrmex* samples that were identified by hierarchical clustering analysis are numbered 1 and 2 as written in the dendrogram, and were each dominated by a single *Pseudonocardia* OTU that was (with only one exception for *A. echinatior*) not found in the other cluster. The tentative placement of these two OTUs in the phylogenetic clades identified by Cafaro *et al*. (2011) is indicated by Roman numbers below our Arabic cluster numbers. At the top of the figure the two *Trachymymex* samples clustered together as they both harboured a unique *Amycolatopsis* OTU. Three of the *A. echinatior* field samples and two of the *Cyphomymex* samples were not placed in either of the two main clusters due to low *Pseudonocardia* prevalence. Both clusters also harboured samples with a higher bacterial diversity (less intense red towards the left and more yellow towards the right with variation increasing from top to bottom in each cluster). The horizontal order of the bacterial OTUs is based on the OTU classification numbers found in the DataS1.

Beta diversity of the samples was analysed by calculating theta values (Yue & Clayton 2005), which allowed comparing the diversity and prevalence of OTUs across samples and resulted in clustering of samples with similar bacterial communities. This was performed in three ways: (i) Including all of the OTUs with the actual number of sequences obtained for each of them; (ii) Including only the 27 OTUs with a prevalence of >5% using the prevalence percentages; and (iii) After removing the 9 OTUs suspected to be external or soft-tissue contaminants (four Pseudomonadales, three Rhizobiales, one Enterobacteriaceae and one Entomoplasmataceae OTU). These three approaches generated very similar results (Fig. 2 and Fig. S2, Supporting information): Two main clusters of *A. echinatior* samples always emerged, each dominated by a different *Pseudonocardia* strain, and with the *Trachymyrmex* samples forming a third cluster. Removing OTUs with a prevalence <5% only affected the placement of sample Ae.406F (from cluster 2 to 1). Removing potential contaminants relocated this sample back to cluster 2, and samples Ae.44F and Ae.480F were moved to a sidebranch of cluster 2 (instead of cluster 1), while Ae.342F was placed in cluster 1 (all of these samples had very low *Pseudonocardia* sequence counts; Table S1, Supporting information). Thus, only the placement of a few field samples was affected by which OTUs were included in the analysis.

In all cases where multiple ants were sampled from the same colony, the individuals carried the same dominant *Pseudonocardia* strain. The only exceptional colony that did not obviously specialize on a single *Pseudonocardia* OTU was represented by the two mature worker samples of Ae.406 that were placed (albeit ambiguously; see above) in cluster 2. These two samples had a relatively low prevalence of the *Pseudonocardia* OTU of cluster 2 (Ps2: 17% and 40%, respectively) but also harboured the *Pseudonocardia* OTU of cluster 1 at an even lower prevalence (4% and 9%). Something similar applied to the *A. volcanus* sample (also a mature worker), which had both *Pseudonocardia* OTUs (at 14% and 61%, respectively, but see Fig. S1B, Supporting information). Sample Ae.342F, on a branch of its own in the dendrogram, was dominated by Enterobacteriaceae and Moraxellaceae (Pseudomonadales) OTUs potentially representing contamination (see above), but the only other bacteria present belonged to the *Pseudonocardia* OTU from cluster 1, suggesting that both field and laboratory samples of this colony harboured similar cuticular communities. Sample Ae.44F had a low prevalence of *Pseudonocardia* at 7%, but the OTU present was that of cluster 2.

Three of the samples from six field colonies previously analysed by Poulsen *et al*. (2005) were assigned to cluster 1 (Ae.47F, Ae.26F, Ae.112F) and two were assigned to cluster 2 (Ae.33F, Ae.24F), which is likely also where Ae.44F belonged (see above). The finding of two *Pseudonocardia* OTUs and the clustering of Ae.33F with Ae.24F, and Ae.47F with Ae.26F and Ae.112F thus replicated the results of Poulsen *et al*. (2005), a study that used the EF-1α gene, corroborating the validity of the two main and largely mutually exclusive *Pseudonocardia* clusters in the Gamboa population of *A. echinatior*.

To further test the observed clustering of samples based on their dominant *Pseudonocardia* OTU, all samples were classified as either Ps1 or Ps2, dependent on the presence of the dominant *Pseudonocardia* OTU. Three *A. echinatior* samples with a *Pseudonocardia* prevalence <8% and Ae.406M and F with two strains were scored as having ‘no dominant strain’. An unweighted UniFrac analysis was then performed in mothur to see whether the clustering in Fig. 2 and Fig. S2, Supporting information could be caused by the dominant *Pseudonocardia* strain, which was found to be highly likely (all three dendrograms, UniFrac significance test: UW score = 1, *P* < 0.001). Also the PCoA analysis showed two clear clusters among the samples in a 3D plot with the unclassified samples scattered in between (Appendix S4, Supporting information).

### Comparing alpha diversity between sample types and clusters

**Figure 3:**
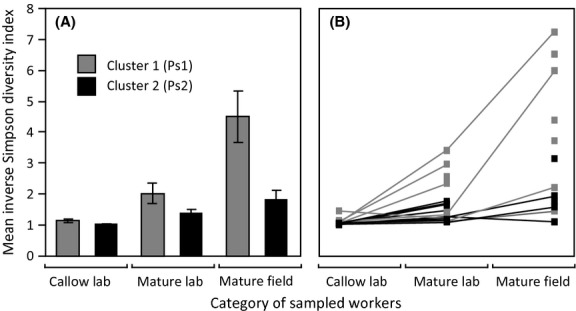
Alpha diversity of bacterial communities. The bacterial diversity estimated with the inverse Simpson index after subsampling of 1023 sequences per sample so that mean ± SE estimates could be obtained. (A) A two-way anova showed that there was a significant diversity difference between clusters Ps1 (grey) and Ps2 (black) and ant-sampling categories (callow, mature, field), and that also the interaction term between these predictor variables was significant (clusters: *F*_1,45_ = 14.89, *P* = 0.0004; ant categories: F_2,45_ = 16.57, *P* < 0.0001; interaction: *F*_2,45_ = 6.74, *P* = 0.003). The mature field worker diversity index of *Pseudonocardia* cluster 1 was significantly higher than the estimate obtained for cluster 2, and field-collected samples had consistently higher diversity than laboratory-collected callow and possibly also mature laboratory workers (see also Fig. 1B and Fig. 2), but the significant interaction term makes the latter result ambiguous. (B) Inverse Simpson diversity index plotted for each individual ant with samples from the same colony connected by lines. A one-way anova with matching columns found no significant effect of colony ID indicating that the differences across colonies are general. See text for further details.

Bacterial alpha diversity from *A. echinatior* cuticles was further analysed by a two-way anova, with the inverse Simpson diversity index as the dependent variable and worker type (callow laboratory workers, mature laboratory workers and mature field workers) and *Pseudonocardia* cluster as factors (only cluster 1 and 2; cf. Fig. 2; sample Ae.406M and *F*, Ae.342F, Ae.480F and Ae.44F were excluded from this analysis). There was an overall difference in the diversity index between the two clusters (*F*_1,45_ = 14.89, *P* = 0.0004), between the three worker categories (*F*_2,45_ = 16.57, *P* < 0.0001) and a significant interaction between these two predictor variables (*F*_2,45_ = 6.74, *P* = 0.003; Fig. 3A). Ants from cluster 1 had a significantly higher diversity, as did workers sampled in the field. The interaction was due to field samples from cluster 1 having the highest diversity, and field samples of cluster 2 not having higher diversity than mature laboratory samples of cluster 1 (Tukey’s HSD, *P* < 0.05, Fig. 3A).

There was a very clear pattern of callow workers having almost exclusively (Inverse Simpson index indistinguishable from 1; Fig. 3A) one of the two *Pseudonocardia* strains that have been inferred to be mutualists in earlier studies (Poulsen *et al*. 2005; Cafaro *et al*. 2011; see also Discussion). The mature workers in the laboratory had slightly but nonsignificantly higher diversity than the callow workers, while field workers had the highest diversity overall. This pattern was also found when taking the colony IDs into account, with colony ID not having an effect (one-way anova with matching columns; category: *F*_2,24_ = 8.09, *P* = 0.003, colony ID: *F*_22,24_ = 1.74, *P* = 0.10; Fig. 3B). There was no significant difference in sequencing depth between the three worker categories (one-way anova, *F*_2,45_ = 0.67; *P* = 0.52), but to minimize any effect of sequencing effort the diversity index was calculated by subsampling 1023 sequences from each sample-specific pool of sequences, which produced no correlation between the alpha diversity index and the total amount of sequences obtained from each sample (Linear regression, *R*^2^ = 0.004, *P* = 0.74).

When only looking at the laboratory-collected samples, there was no correlation between how long the ants had been kept in the laboratory (measured as years since collection) and their cuticular bacterial alpha diversity (Linear Regression, *R*^2^ = 0.0036, *P* = 0.74), suggesting that the extent of the laboratory tenure does not affect the diversity of cuticular bacterial communities. Eleven of the *Acromyrmex* colonies from the laboratory that were assigned to either cluster 1 or 2 had been kept in climate rooms with primarily other *Acromyrmex* and *Atta* leaf-cutting ants (room ‘Acro 3’ and room ‘Atta’, Table S1, Supporting information), whereas eight had been kept together with a variety of lower attine ants (such as *Apterostigma*, *Myrmicocrypta*, *Mycocepurus*, *Cyphomyrmex*) and higher attine but nonleafcutter ants of the genera *Trachymyrmex* and *Sericomyrmex* (room ‘Acro 1’ and room ‘Q’; Table S1, Supporting information). However, there was no significant correlation between which room the colony had been kept in and which cluster its bacterial community belonged to (Likelihood ratio test, *χ*^2^_1,19_ = 2.87, *P* = 0.09). This suggests that having neighbouring colonies with different actinomycetes for up to several years does not by itself increase the probability of horizontal acquisition, consistent with all colonies for which we had both field and laboratory samples retaining their dominant *Pseudonocardia* OTU.

## Discussion

### Bacterial diversity on the cuticle of leaf-cutting ants in Gamboa, Panama

Our analyses of the diversity of bacterial communities identified two *Pseudonocardia* strains that dominated the cuticular diversity of *Acromyrmex echinatior*, and these almost never co-occurred in the same colony or on the same ants (the only exception being the two samples from Ae.406; Fig. 2). The association between *A. echinatior* colonies and their *Pseudonocardia* strain was very stable, with 10 of 10 colonies that had been kept in the laboratory for up to 10 years in each other’s close proximity retaining their original strains, in spite of potential opportunities for horizontal transmission from neighbouring colonies. The colonies used in the present study had been collected from the same field site over a period of 17 years with both strains being sampled across that period, confirming the stable co-occurrence of both strains in the Gamboa *Acromyrmex* population. Analysis of less conserved genes than the 16S rRNA region targeted here may reveal a greater diversity within and between colonies, but sequencing of the more variable gene EF-1α after culturing *Pseudonocardia* sampled from ant cuticles also found just two strains on *A. echinatior* and its sister species *A. octospinosus* in the study area (Poulsen *et al*. 2005). We therefore consider the results of the two combined studies to be robust evidence for a long-term association between *Acromyrmex* colonies and normally a single vertically transmitted strain of *Pseudonocardia*.

Other Actinomycetales bacteria were found to be inconsistently associated with the mesosomal cuticles of the attine ants that we analysed. In 21 samples (11 from the field and 10 from laboratory colonies, all mature workers), six OTUs of other Actinobacteria reached a cumulative prevalence of 5–41% (not considering prevalences <5%). Varying densities of *Pseudonocardia* bacteria were also found in these samples, but without estimates of the total bacterial density, it was not possible to infer whether the densities of these vertically acquired *Pseudonocardia* were negatively correlated with densities of other Actinomycetales, as would be expected when strains compete (Scheuring & Yu 2012). Among these other Actinomycetales, four putative *Streptomyces* OTUs were observed at prevalences of 5–29% in 17 samples of mature ants, 11 of which were field collected. *Streptomyces* has previously been isolated from both *A. echinatior* and its sympatric congeners *A. octospinosus* and *A. volcanus*, from both workers and fungus gardens in different geographical locations (Kost *et al*. 2007; Haeder *et al*. 2009; Barke *et al*. 2010; Schoenian *et al*. 2011; Seipke *et al*. 2011). The *Streptomyces* found associated with *Acromyrmex* have been shown to produce distinct antibiotics on the ant cuticle (Schoenian *et al*. 2011) and were hypothesized to be regularly acquired from the soil as complementary sources of antibiotics to the resident *Pseudonocardia* bacteria (Barke *et al*. 2010). It is important to note, however, that all large worker ants carrying *Streptomyces* were older foragers, consistent with these bacteria not having a role in defence against *Escovopsis*, in contrast to the callow workers that have not yet left the nest (see also next section).

### Community diversity across life stages and environments

No difference was found in the overall bacterial diversity between callow and mature workers in the laboratory, but it is worth noting that, while not significantly different from mature workers, the callow workers had a diversity index very close to one, meaning almost complete dominance of a single *Pseudonocardia* strain. At the sampled stage, the callow workers were completely covered in bacterial growth and resided in the fungus garden and we thus infer that it is especially at this stage that the large workers are important for defence against *Escovopsis* infections. Experimental evidence for this contention was obtained by Currie *et al*. (2003b) for sympatric Panamanian *A. octospinosus*, which is known to share the two main *Pseudonocardia* strains with *A. echinatior* at the Gamboa sampling site (Poulsen *et al*. 2005).

The field samples had a significantly higher diversity than the laboratory samples. It seems likely that field ants are exposed to a greater diversity of additional bacteria that could potentially grow on their cuticle. Finding this higher diversity in field samples, owing in considerable measure to the presence of other Actinobacteria on the cuticle of mature field workers, is consistent with the modelling scenario developed by Scheuring & Yu (2012): Vertically transmitted *Pseudonocardia* bacteria dominating on callow workers that are confined to the inside of the colony where fungus-garden infections may strike, and older foragers acquiring additional environmental strains later in their lives. The mean diversity index of cluster 1 field samples was higher than that of cluster 2 field samples (Fig. 3A). It would thus be interesting to obtain a larger set of field and laboratory samples across multiple colonies to test with greater statistical power whether the two *Pseudonocardia* OTUs may differ in their ability to continue growing in pure culture on older workers.

Horizontal transmission of *Pseudonocardia* between colonies and species under laboratory conditions could not be demonstrated in our present study, as the field samples obtained in the second 454 run showed that all colonies for which we had both field and laboratory samples retained their characteristic bacterial community. Taken together, the implication of our findings is that *A. echinatior* colonies with communities dominated by one of their two vertically acquired *Pseudonocardia* strains are apparently capable of maintaining their original *Pseudonocardia* symbiont even when kept in close proximity to colonies having other bacteria on their cuticle and that the original *Pseudonocardia* strain always appears in approximate monoculture in newly hatched callow workers (Figs 2 and 3). We therefore feel it is justified to consider these two strains as native mutualists that are obligatorily associated with *A. echinatior* in Gamboa, Panama.

To further address the potential for inter-specific horizontal transmission of bacteria in the laboratory, four other species of attine ants were included in the analysis. The *Trachymyrmex* species carried an entirely different genus of Actinobacteria, *Amycolatopsis*, and a low diversity of Actinobacteria was found on *C. longiscapus*. There was extensive overlap in the actinomycete communities of *A. echinatior* and *C. costatus*, which were carrying the *Pseudonocardia* OTU from cluster 1, but it was not possible to evaluate whether this had also been the case in the field. *A. volcanu*s and colony Ae.406 were interesting in apparently harbouring two different *Pseudonocardia* OTUs, in both cases dominated by the OTU from cluster 2. However, classification of OTUs with a stricter 99% similarity cut-off suggested that for *A. volcanus,* these results should be interpreted with some caution as this sample might also have carried a monoculture of a distinct *Pseudonocardia* strain (Fig. S1B, Supporting information). *A. volcanus* has only rarely been encountered at the study site and is typically found nesting in wood cavities in the canopy. The mature foraging workers are clearly distinct from those of *A. echinatior* and *A. octospinosus* by being almost black and covered in white bacterial bloom also at a more advanced age. How and why this growth is sustained remains unclear, but maintaining high degrees of bacterial cover throughout adult life may well change the bacterial interactions on the ant cuticle. The presence of both *Pseudonocardia* OTUs in a 4:1 ratio in the field and the laboratory sample from colony Ae.406 (sequenced in two separate runs) suggests that this exceptional association may be stable over time. So, in spite of most colonies being characterized by a single native *Pseudonocardia* strain, possible exceptions like this will merit further investigation.

The potential for colony-level adaptation to specific cuticular bacterial communities was not directly addressed in this study, but our results indicate that this remains a realistic possibility. The *c*. 50:50 distribution of the two *Pseudonocardia* strains in the Gamboa population across many years suggests that some form of balancing selection may act on the *Pseudonocardia* community composition. Cross-fostering experiments have recently shown that native *Pseudonocardia* bacteria grow less well on newly eclosed ants developing in another colony than their own (Armitage *et al*. 2011), and it has subsequently been confirmed that the dominant *Pseudonocardia* of these colonies belonged to cluster 1 and 2, respectively (Andersen *et al*. in prep). This suggests that the unknown glandular secretions that feed the native *Pseudonocardia* strains may have some degree of co-adaptation to harbouring either Ps1 or Ps2. Further work will be needed to clarify if this is the case and whether that might explain why the likelihood of foragers in the field having their cuticles colonized by other bacteria may be higher for workers carrying Ps1 rather than Ps2 (Fig. 3).

## Conclusions

In the present study, we exploited the potential of high-throughput sequencing to obtain diversity estimates of bacterial communities from the cuticle of Panamanian *Acromyrmex* leaf-cutting ants. We show that diversity per colony is relatively low and revolves around a single dominant and vertically transmitted strain of *Pseudonocardia*. These results are consistent with earlier findings by Poulsen *et al*. (2005) based on single-gene sequencing of ant-derived *Pseudonocardia* cultures. This implies that the *Acromyrmex–Pseudonocardia* symbiosis in our study population combines intriguing characteristics of long-term interaction specificity with indications that horizontal acquisition of other Actinobacteria may indeed happen and gradually increase when workers become more active outside the nest. Our data indicate that the two *Pseudonocardia* strains originally identified as being shared between *A. echinatior* and *A. octospinosus* in Gamboa, Panama, are long-term associated native mutualists of the *Acromyrmex* population that we studied and that they are predictably present on the callow workers that are most likely to have defence functions against *Escovopsis*. Our findings are consistent with both the assumptions and predictions of the model developed by Scheuring & Yu (2012) to address questions of coexistence and stability of mutualistic function in actinomycete communities on attine ant cuticles.

## Notes

S.B. Andersen and J.J. Boomsma designed the study, S.B. Andersen and P. Sapountzis did the experimental work, L.H. Hansen and S.J. Sørensen contributed primers, tags, adaptors, and reagents for 454 pyrosequencing, and assisted with library preparation and interpretation of the sequencing results, S.B. Andersen performed the analyses, and S.B. Andersen and J.J. Boomsma wrote the paper.

## Data accessibility

DNA sequences: NCBI SRA: SRA050635. Representative DNA sequences for described OTUs: uploaded as online Supporting Information. Alignment of representative sequences for all OTUs, read distribution for all OTUs across samples, OTU classification list, representative sequences for all OTUs and the scripts used for data analyses in mothur: deposited in Dryad entry doi:10.5061/dryad.c4f03.

## Supporting Information

Fig S1 Number and classification of sequences obtained from the cuticles of large workers of *Acromyrmex echinatior*.

Fig S2 Dendrograms showing clustering of samples following additional analyses with the UPGMA algorithm in mothur.

Table S1 Description of the ant samples used. **Table S2** Phylogenetic distribution of the 27 OTUs that had a prevalence of >5% in at least one sample.

Appendix S1 A FASTA file with the representative sequences for the 27 most abundant OTUs.

Appendix S2 A FASTA with the representative sequences for the five Ps1 OTUs identified with a 99% similarity cut-off.

Appendix S3 FASTA files containing all 454 reads identified as Ps1 with a 97% similarity cut-off for six selected samples.

Appendix S4 Video of a 3D plot of the first three principal coordinates for each sample following a Principle Coordinates Analysis (PCoA).
