# Supplementary figures and images for: Specificity and stability of the Acromyrmex–Pseudonocardia symbiosis
Source: Mol Ecol. 2013 Jul 30;22(16):4307–21. doi: 10.1111/mec.12380 (PMC4228762; doi:10.1111/mec.12380)

Fig. S1

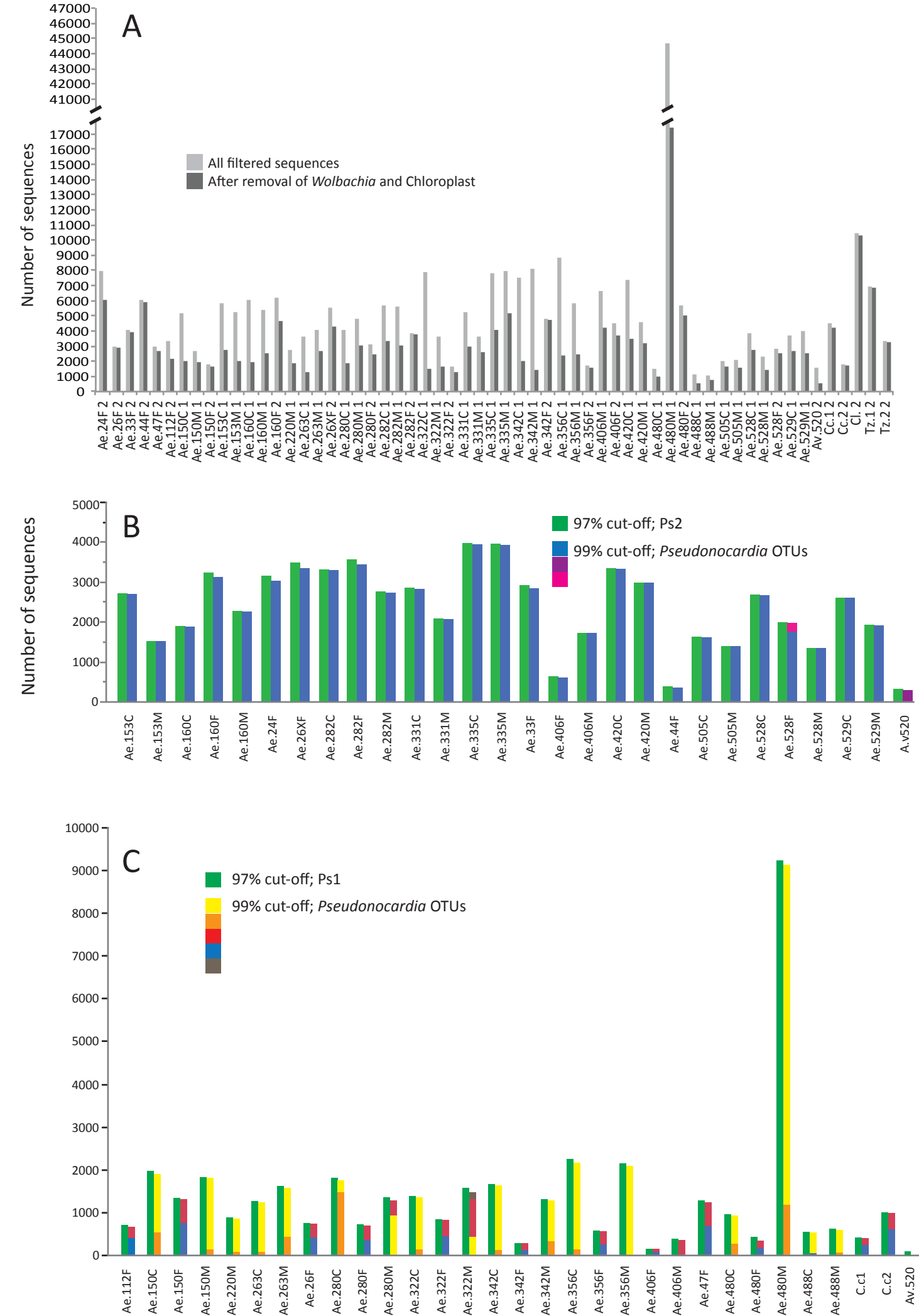

Supplement: Fig S1 — Number and classification of sequences obtained from the cuticles of large workers of Acromyrmex echinatior. [file mec0022-4307-sd1.pdf]

All OTUs

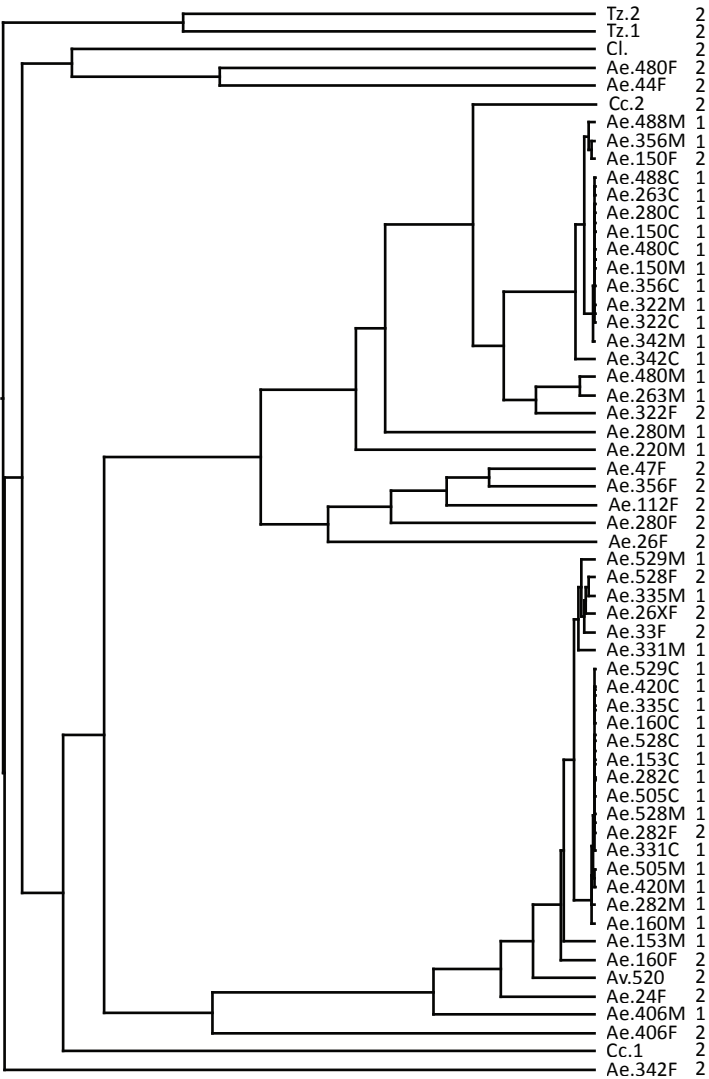

19 OTUs  
with suspected contaminants removed

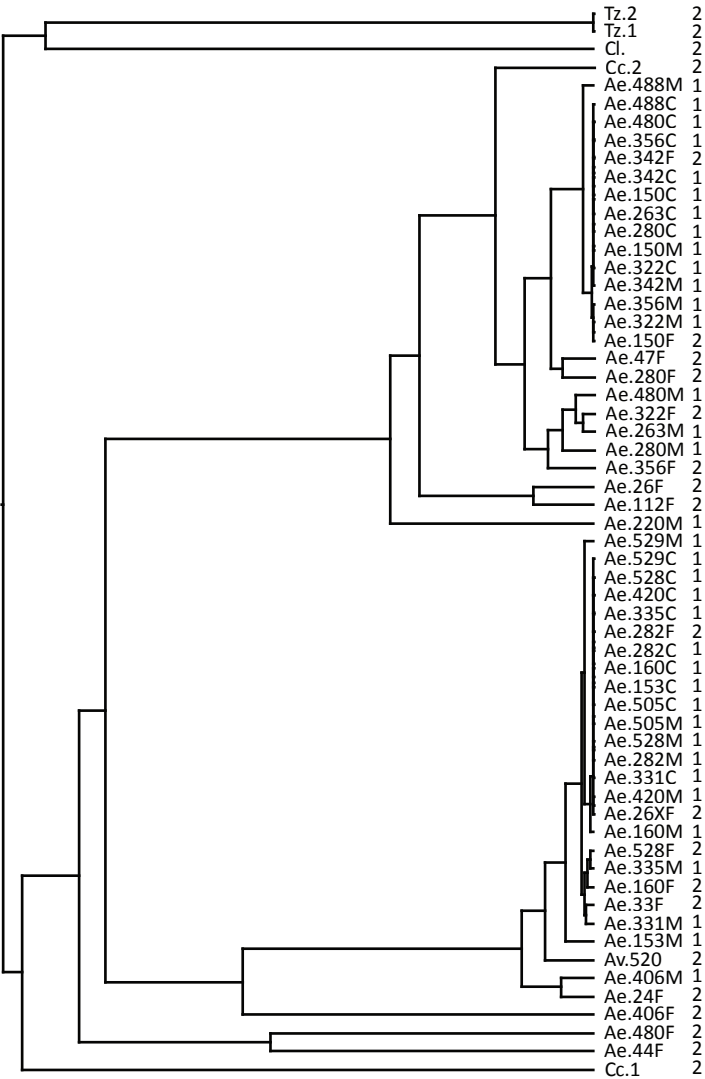

Supplement: Fig S2 — Dendrograms showing clustering of samples following additional analyses with the UPGMA algorithm in mothur. [file mec0022-4307-sd2.pdf]
