# Supplementary material for: Specificity and stability of the Acromyrmex–Pseudonocardia symbiosis
Source: Mol Ecol. 2013 Jul 30;22(16):4307–21. doi: 10.1111/mec.12380 (PMC4228762; doi:10.1111/mec.12380)
Supplement: Table S1 — Description of the ant samples used. Table S2 Phylogenetic distribution of the 27 OTUs that had a prevalence of >5% in at least one sample. [file mec0022-4307-sd3.docx]

**Supplementary Tables**

**Table S1**

| **Sample ID** | **Collection year** | **Name of ant room in the lab** | **Sequencing run** | **Number of sequences** | **Number of sequences**  **after removing *Wolbachia* and chloroplast sequences** | **Number of Ps1 sequences** | **Number of Ps2 sequences** |
| --- | --- | --- | --- | --- | --- | --- | --- |
| Ae.24F | 1994 | **-** | 2 | 7930 | 6045 |  | 3156 |
| Ae.26F | 1994 | **-** | 2 | 2947 | 2874 | 756 |  |
| Ae.33F | 1996 | **-** | 2 | 4068 | 3907 |  | 2921 |
| Ae.44F | 1996 | **-** | 2 | 6073 | 5882 |  | 384 |
| Ae.47F | 1996 | **-** | 2 | 2949 | 2688 | 1280 |  |
| Ae.112F | 2000 | **-** | 2 | 3359 | 2200 | 711 |  |
| Ae.150F | 2001 | - | 2 | 1789 | 1636 | 1352 |  |
| Ae.150C | 2001 | Atta | 1 | 5195 | 2052 | 1977 |  |
| Ae.150M | 2001 | Atta | 1 | 2686 | 1936 | 1831 |  |
| Ae.153C | 2001 | Atta | 1 | 5867 | 2766 |  | 2719 |
| Ae.153M | 2001 | Atta | 1 | 5282 | 1989 |  | 1522 |
| Ae.160F | 2002 | - | 2 | 6203 | 4688 |  | 3236 |
| Ae.160C | 2011 | Acro 1 | 1 | 6071 | 1957 |  | 1899 |
| Ae.160M | 2011 | Acro 1 | 1 | 5365 | 2568 |  | 2278 |
| Ae.220M | 2004 | Acro 3 | 1 | 2753 | 1869 | 886 |  |
| Ae.263C | 2004 | Acro 3 | 1 | 3667 | 1308 | 1270 |  |
| Ae.263M | 2011 | Acro 3 | 1 | 4060 | 2668 | 1622 |  |
| Ae.26XF | 2004 | - | 2 | 5572 | 4289 |  | 3486 |
| Ae.280F | 2004 | - | 2 | 3134 | 2463 | 729 |  |
| Ae.280C | 2011 | Acro 3 | 1 | 4037 | 1893 | 1814 |  |
| Ae.280M | 2011 | Acro 3 | 1 | 4841 | 3073 | 1355 |  |
| Ae.282F | 2004 | - | 2 | 3871 | 3757 |  | 3570 |
| Ae.282C | 2011 | Acro 1 | 1 | 5693 | 3357 |  | 3306 |
| Ae.282M | 2011 | Acro 1 | 1 | 5592 | 3059 |  | 2760 |
| Ae.322F | 2006 | - | 2 | 1677 | 1310 | 851 |  |
| Ae.322C | 2011 | Acro 3 | 1 | 7906 | 1495 | 1396 |  |
| Ae.322M | 2011 | Acro 3 | 1 | 3643 | 1677 | 1576 |  |
| Ae.331C | 2007 | Acro 1 | 1 | 5247 | 2994 |  | 2852 |
| Ae.331M | 2011 | Acro 1 | 1 | 3634 | 2572 |  | 2080 |
| Ae.335C | 2007 | Atta | 1 | 7845 | 4091 |  | 3976 |
| Ae.335M | 2011 | Atta | 1 | 7931 | 5165 |  | 3956 |
| Ae.342F | 2007 | - | 2 | 4784 | 4715 | 229 |  |
| Ae.342C | 2011 | Acro 3 | 1 | 7550 | 2041 | 1665 |  |
| Ae.342M | 2011 | Acro 3 | 1 | 8140 | 1450 | 1321 |  |
| Ae.356F | 2008 | - | 2 | 1691 | 1598 | 577 |  |
| Ae.356C | 2011 | Acro 3 | 1 | 8864 | 2401 | 2254 |  |
| Ae.356M | 2011 | Acro 3 | 1 | 5807 | 2476 | 2146 |  |
| Ae.406F | 2009 | - | 2 | 4536 | 3734 | 157 | 638 |
| Ae.406M | 2011 | Acro 3 | 1 | 6632 | 4232 | 385 | 1728 |
| Ae.420C | 2009 | Acro 3 | 1 | 7382 | 3469 |  | 3353 |
| Ae.420M | 2011 | Acro 3 | 1 | 4605 | 3216 |  | 2988 |
| Ae.480F | 2010 | - | 2 | 5674 | 5049 | 429 |  |
| Ae.480C | 2011 | Acro 1 | 1 | 1529 | 1023 | 967 |  |
| Ae.480M | 2011 | Acro 1 | 1 | 44716 | 17456 | 9232 |  |
| Ae.488C | 2010 | Acro 1 | 1 | 1114 | 577 | 558 |  |
| Ae.488M | 2011 | Acro 1 | 1 | 1060 | 760 | 625 |  |
| Ae.505C | 2011 | Q | 1 | 1987 | 1665 |  | 1625 |
| Ae.505M | 2011 | Q | 1 | 2060 | 1546 |  | 1399 |
| Ae.528F | 2011 | - | 2 | 2793 | 2566 |  | 1988 |
| Ae.528C | 2011 | Q | 1 | 3825 | 2726 |  | 2678 |
| Ae.528M | 2011 | Q | 1 | 2314 | 1410 |  | 1352 |
| Ae.529C | 2011 | Q | 1 | 3671 | 2714 |  | 2612 |
| Ae.529M | 2011 | Q | 1 | 4014 | 2542 |  | 1922 |
| Av.520 | 2011 | Q | 2 | 1551 | 551 | 81 | 339 |
| Tz.1  _(15-02 2010-2)_ | 2010 | Q | 2 | 6919 | 6833 |  |  |
| Tz.2  _(022-0509)_ | 2009 | Acro 1 | 2 | 3326 | 3251 |  |  |
| Cc.1  _(RMMA100603-04)_ | 2004 | Q | 2 | 4490 | 4219 | 421 |  |
| Cc.2  _(011-170507)_ | 2007 | Acro 1 | 2 | 1782 | 1759 | 1010 | 104 |
| Cl. | 2006? | Acro 1 | 2 | 10463 | 10341 |  |  |

**Table S2**

| **Phylum** | **Order / Family / Genus** | **Number of OTUs** | **Mean % contribution per sample ± SE** |
| --- | --- | --- | --- |
| Actinobacteria | *Pseudonocardia* | 2 | 69 ± 4% |
|  | *Streptomyces (?)* | 4 | 4 ± 1% |
|  | Nocardioidaceae | 1 | 3 ± 1% |
|  | *Amycolatopsis* | 1 | 2 ± 2% |
|  | Solirubrobacterales | 1 | 0 ± 0% |
| Proteobacteria | Rhizobiales | 3 | 3 ± 2% |
|  | Pseudomonadales | 4 | 4 ± 1% |
|  | Xanthomonadaceae | 2 | 2 ± 1% |
|  | Enterobacteriaceae Burkholderiales  Sphingomonadaceae | 1  1  1 | 1 ± 1%  1 ± 0%  0 ± 0% |
| Bacteroidetes | Chitinophagaceae (?) | 2 | 1 ± 0% |
| Tenericutes | Entomoplasmataceae | 1 | 2 ± 0% |
| Acidobacteria | Unclassified | 2 | 0 ± 0% |
| Firmicutes | Clostridiales | 1 | 0 ± 0% |
