# Supplementary material for: Specificity and stability of the Acromyrmex–Pseudonocardia symbiosis
Source: Mol Ecol. 2013 Jul 30;22(16):4307–21. doi: 10.1111/mec.12380 (PMC4228762; doi:10.1111/mec.12380)
Supplement: Appendix S1 — A FASTA file with the representative sequences for the 27 most abundant OTUs. [file mec0022-4307-sd4.rtf]

>Ps1TGGGGAATATTGCGCAATGGGCGGAAGCCTGACGCAGCGACGCCGCGTGGGGGATGACGGCCTTCGGGTTGTAAACCTCTTTCGCCAGGGACGAAGCTTTTGTGACGGTACCTGGAGAAGAAGCACCGGCCAACTACGTGCCAGCAGCCGCGGTAACACGTAGGGTGCGAGCGTTGTCCGGAATTATTGGGCGTAAAG>Ps2TGGGGAATATTGCGCAATGGGCGGAAGCCTGACGCAGCGACGCCGCGTGGGGGATGACGGCCTTCGGGTTGTAAACCTCTTTCGCCAGGGACGAAGAGTGATTGACGGTACCTGGAGAAGAAGCACCGGCCAACTACGTGCCAGCAGCCGCGGTAACACGTAGGGTGCGAGCGTTGTCCGGAATTATTGGGCGTAAAG>OTU003TTGGGAATATTGCACAATGGGCGCAAGCCTGATGCAGCGACGCCGCGTGAGGGATGACGGCCTTCGGGTTGTAAACCTCTTTCAGTAGGGAAGAAGCGAAAGTGACGGTACCTGCAGAAGAAGCACCGGCTAACTACGTGCCAGCAGCCGCGGTAATACGTAGGGTGCAAGCGTTGTCCGGAATTATTGGGCGTAAAG>OTU004TGGGGAATATTGGACAATGGGCGAAAGCCTGATCCAGCCATGCCGCGTGTGTGAAGAAGGTCTTCGGATTGTAAAAGCACTTTAAGTTGGGAGGAAGGGCAGTAAATTAATACTTTGCTGTTTTGACGTTACCGACAGAATAAGCACCGGCTAACTCTGTGCCAGCAGCCGCGGTAATACAGAGGGTGCAAGCGTTAATCGGAATTACTGGGCGTAAAG>OTU006TAGGGAATTTTTCACAATGGACGAAAGTCTGATGAAGCAATGCCGCGTGAGTGATGAAGGTCTTCGGATTGTAAAGCTCTGTTGTAAGGGAAGAATAAGCCAGGAAGGAAATGTCTTGGTGATGACGGTACCTTACCAGAAAGCCCCGGCTAACTATGTGCCAGCAGCCGCGGTAATACATAGGGGGCAAGCGTTATCCGGATTTATTGGGCGTACAG>OTU008TGGGGAATATTGGACAATGGGCGGAAGCCTGATCCAGCCATGCCGCGTGAGTGAAGAAGGCCTTAGGGTTGTAAAAGCTCTTTCACCGGTGAAGATAATGACGGTAGCCGGAGAAGAAGCCCCGGCTAACTTCGTGCCAGCAGCCGCGGTAATACGAAGGGGGCTAGCGTTGTTCGGATTTACTGGGCGTAAAG>OTU023TGGGGAATATTGGACAATGGGCGAAAGCCTGATCCAGCAATGCCGCGTGAGTGATGAAGGCCTTAGGGTTGTAAAGCTCTTTTACCCGGGATGATAATGACAGTACCGGGAGAATAAGCTCCGGCTAACTCCGTGCCAGCAGCCGCGGTAATACGGAGGGAGCTAGCGTTGTTCGGAATTACTGGGCGTAAAG>OTU027TGGGGAATATTGGACAATGGGCGAAAGCCTGATCCAGCCATGCCGCGTGTGTGAAGAAGGCCTTTTGGTTGTAAAAGCACTTTAAGCGAGGAGGAGGCTACTTAGATTAATACTCTAGGATAGTGGACGTTACTCGCAGAATAAGCACCGGCTAACTCTGTGCCAGCAGCCGCGGTAATACAGAGGGTGCGAGCGTTAATCGGATTTACTGGGCGTAAAG>OTU036TGGGGAATATTGCACAATGGGCGAAAGCCTGATGCAGCGACGCCGCGTGAGGGATGACGGCCTTCGGGTTGTAAACCTCTTTCAGCAGGGACGAAGCGAAAGTGACGGTACCTGCAGAAGAAGCGCCGGCCAACTACGTGCCAGCAGCCGCGGTAATACGTAGGGCGCAAGCGTTGTCCGGAATTATTGGGCGTAAAG>OTU037TGGGGAATATTGGACAATGGGCGAAAGCCTGATCCAGCAACGCCGCGTGAGGGATGACGGCCTTCGGGTTGTAAACCTCTTTCAGCACCGACGAAGCGAAAGTGACGGTAGGTGCAGAAGAAGGACCGGCCAACTACGTGCCAGCAGCCGCGGTAATACGTAGGGTCCGAGCGTTGTCCGGAATTATTGGGCGTAAAG>OTU038TAGGGAATATTGCACAATGGGCGAAAGCCTGATGCAGCGACGCCGCGTGAGGGATGACGGCCTTCGGGTTGTAAACCTCTTTCAGCAGGGAAGAAGCGAAAGTGACGGTACCTGCAAAAGAAGCACCGGCTAACTACGTGCCAGCAGCCGCGGTAATACGTAGGGTGCGAGCGTTGTCCGGAATTATTGGGCGTAAAG>OTU039TAAGGAATATTGGTCAATGGAGGAAACTCTGAACCAGCCATGCCGCGTGGAGGATGAAGGTCCTCTGGATTGTAAAACTTCTTTTATCTGGGACGAAAAAAGGGAATTCTTTCCCGACTGACGGTACCAGATGAATAAGCACCGGCTAACTCCGTGCCAGCAGCCGCGGTAATACGGAGGGTGCAAGCGTTATCCGGATTCACTGGGTTTAAAG>OTU041TGGGGAATATTGCACAATGGGCGGAAGCCTGATGCAGCGACGCCGCGTGAGGGATGACGGCCTTCGGTTGTAAACCTCTTTCAGCAGGGAAGAAGCTTTTGTGACGGTACCTGCAGAAGAAGCACCGGCTAACTACGTGCCAGCAGCCGCGGTAATACGTAGGGTGCGAGCGTTGTCCGGAATTATTGGGCGTAAAG>OTU042TGGGGAATATTGGACAATGGGGGGAACCCTGATCCAGCCATGCCGCGTGTGTGAAGAAGGCCTTTTGGTTGTAAAGCACTTTAAGTGGGGAGGAGGCTCTCTTGGTTAATACCTAAGAAGAGTGGACGTTACCCACAGAATAAGCACCGGCTAACTCTGTGCCAGCAGCCGCGGTAATACAGAGGGTGCGAGCGTTAATCGGATTTACTGGGCGTAAAG>OTU043TAGGGAATCTTGCGCAATGCGCGAAAGCGTGACGCAGCAACGCCGCGTGGGGGAAGACGGTCTTCGGATTGTAAAACCCCTTTCAGTTGGGACGAAGCTTCGCCGGTTAATAGCCGGCTGGAGTGACGGTACCTTCACAAGAAGCCCCGGCTAACTACGTGCCAGCAGCCGCGGTAATACGTAGGGGGCGAGCGTTGTCCGGAATCATTGGGCGTAAAG>OTU046TAGGGAATTTTGGACAATGGGCGAAAGCCTGATCCAGCAATGCCGCGTGTGTGATGAAGGCCTTCGGTTGTAAAGCACTTTTGGTAGGAACGAAATGGTCCGTGCTAATACCATGGATCGATGACGGTACCTGCAGAATAAGCACCGGCTAACTACGTGCCAGCAGCCGCGGTAATACGTAGGGTGCAAGCGTTAATCGGAATTACTGGGCGTAAAG>OTU065TGGGGAATATTGGACAATGGGCGAAAGCCTGATCCAGCCATGCCGCGTGAGTGATGAAGGCCCTAGGGTTGTAAAGCTCTTTCAACGGTGAAGATAATGACGGTAACCGTAGAAGAAGCCCCGGCTAACTTCGTGCCAGCAGCCGCGGTAATACGAAGGGGGCTAGCGTTGTTCGGATTTACTGGGCGTAAAG>OTU072TGGGGAATTGTTCACAATGGGCGCAAGCCTGATGACGCAACGCCGCGTGGGTGATGAAGGTCTTCGGATTGTAAAACCCCTGTCGAAATGGGACGAAAAGACTCGCGAGTTAATACTCCGCGAGAGTGACGGTACCGTTAAAAGGAAGCCACGGCTAACTCTGTGCCAGCAGCCGCGGTAATACAGAGGTGGCAAGCGTTGTTCGGAATTACTGGGCTAAAG>OTU113TGGGGAATATTGGACAATGGGGGGAACCCTGATCCAGCCATGCCGCGTGTGTGAAGAAGGCCTTTTGGTTGTAAAAGCACTTTTAAGCGAGGAGGAGGCTACCGAGATTAATACTCTTGGATAGTGGACGTTACTCGCAGAATAAGCACCGGCTAACTCTGTGCCAGCAGCCGCGGTAATACAGAGGGTGCAAGCGTTAATCGGATTTACTGGGCGTAAAG>OTU126TGGGGAATATTGCACAATGGGGGAAACCCTGATGCAGCGACGCCGCGTGAGTGAAGAAGTATTTCGGTATGTAAAAGCTCTATCAGCAGGGAAGAAAGTGACGGTACCTGAATAAGAAGCCCCGGCTAACTACGTGCCAGCAGCCGCGGTAATACGTAGGGGGCAAGCGTTATCCGGATTTACTGGGTGTAAAG>OTU182TGGGGAAGTAATTGGACAATGGGCGAAAGCCTGATCCAGCCATGCCGCATGAGTGAAGAAGGCCTTCGGGTTGTAAAGACTCTTTTAGTTGAGGAAGATAATGACGGTACTCACAGAAGAAGTCCTGGCTAACTCCGTGCCAGCAGCCGCGGTAATACGGAGAGGGCTAGCGTTATTCGGAATCATTGGGCGTAAAG>OTU233TAGGGAATATTGGACAATGGGCGCAAGCCTGATCCAGCCATGCCGCGTGAGTGAAGAAGGCCCTCGGGTTGTAAAAGCTCTTTTGTCCGGAAAGAAAAGCACTTGGCTAATACCCAGGTGTCCTGACGGTACCGGAAGAATAAGCACCGGCTAACTTCGTGCCAGCAGCCGCGGTAATACGAAGGGTGCAAGCGTTACTCGGAATTACTGGGCGTAAAG>OTU255TGGGGAATATTGCACAATGGGCGAAAGCCTGATGCAGCGACGCCGCGTGAGGGATGACGGCCTTCGGTTGTAAACTTCTTTCGCCAGGGACGAAGCGCAAGTGACGGTACCTGGATAAGAAGCACCGGCTAACTACGTGCCAGCAGCCGCGGTAATACGTAGGGTGCGAGCGTTGTCCGGAATTATTGGGCGTAAAG>OTU259TAAGGGAATATTGGTCAATGGAGGGAACTCTGAACCAGCCATGCCGCGTGGAGGATGAAGGTCCTCTGGATTGTAAAACTTCTTTTATCTGGGAAGAAACGGGTCTTATCTAAGATTTTTGACGGTACCAGAAGAATAAGCACCGGCTAACTCCGTGCCAGCAGCCGCGGTAATACGGAGGGTGCAAGCGTTATCCGGATTCACTGGGTTTAAAG>OTU521TGGGGAATATTGCACAATGGGCGCAAGCCTGATGCAGCCATGCCGCGTGTATGAAGAAGGCCTTCGGGTTGTAAAGTACTTTCAGCGAGGAGGAAGGCGTTAAGGTTAATAACCTTAGCGATTGACGTTACTCGCAGAAGAAGCACCGGCTAACTCCGTGCCAGCAGCCGCGGTAATACGGAGGGTGCAAGCGTTAATCGGAATTACTGGGCGTAAAG>OTU523TAGGGAATATTGGACAATGGGCGCAAGCCTGATCCAGCCATGCCGCGTGAGTGAAGAAGGCCCTCGGGTTGTAAAAGCTCTTTTGTCCGGAAAGAAAAGCCTCTGGTTAATACCCAGATGTCCTGACGGTACCGGAAGAATAAGCACCGGCTAACTTCGTGCCAGCAGCCGCGGTAATACGAAGGGTGCAAGCGTTACTCGGAATTACTGGGCGTAAAG>OTU893TGGGGAATTTTGCGCAATGGGGGAAACCCTGACGCAGCAACGCCGCGTGGAGGATGAAGTCCCTTGGGACGTAAACTCCTTTCGACCCGGACGATAATGACGGTACGGGTGGAAGAAGCCCCGGCTAACTTCGTGCCAGCAGCCGCGGTAATACGAGGGGGGCAAGCGTTGTTCGGAATTATTGGGCGTAAAG>WolbachiaTGGGGAATATTGCGCAATGGGCGAAAGCCTGATCCAGCCATGCCGCATGAGTGAAGAAGGCCTTCGGGTTGTAAAGCTCTTTTTAGTGAGGAAGATAATGACGGTACTCACAGAAGAAGTCCTGGCTAACTCCGTGCCAGCAGCCGCGGTAATACGGAGAGGGCTAGCGTTATTCGGAATCATTGGGCGTAAAG
