# Supplementary material for: Specificity and stability of the Acromyrmex–Pseudonocardia symbiosis
Source: Mol Ecol. 2013 Jul 30;22(16):4307–21. doi: 10.1111/mec.12380 (PMC4228762; doi:10.1111/mec.12380)
Supplement: Appendix S2 — A FASTA with the representative sequences for the five Ps1 OTUs identified with a 99% similarity cut-off. [file mec0022-4307-sd5.rtf]

>OTU1(A.vol)TGGGGAATATTGCGCAATGGGCGGAAGCCTGACGCAGCGACGCCGCGTGGGGGATGACGGCCTTCGGTTGTAAACCTCTTTCGCCAGGGACGAAGAGCAATTGACGGTACCTGGAGAAGAAGCACCGGCCAACTACGTGCCAGCAGCCGCGGTAACACGTAGGGTGCGAGCGTTGTCCGGAATTATTGGGCGTAAAG>OTU61TGGGGAATATTGCGCAATGGGCGGAAGCCTGACGCAGCGACGCCGCGTGGGGGATGACGGCCTTCGGTTGGTAAACCTCTTTCGCCAGGGACGAAGCTTTTGTTGACGGTACCTGGAGAAGAAGCACCGGCCAACTACGTGCCAGCAGCCGCGGTAACACGTAGGGTGCGAGCGTTGTCCGGAATTATTGGGCGTAAAG>OTU62TGGGGAATATTGCGCAATGGGCGGAAGCCTGACGCAGCGACGCCGCGTGGGGGATGACGGCCTTCGGTTGTAAACCTCTTTCGCCAGGGACGAAGCTTTTGTTGACGGTACCTGGAGAAGAAGCACCGGCCAACTACGTGCCAGCAGCCGCGGTAACACGTAGGGTGCGAGCGTTGTCCGGAATTATTGGGCGTAAAG>OTU161TGGGGAATATTGCGCAATGGGCGGAAGCCTGACGCAGCGACGCCGCGTGGGGGATGACGGCCTTCGGTTGTAAACCTCTTTCGCCAGGGACGAAGCTTTTGTGACGGTACCTGGAGAAGAAGCACCGGCCAACTACGTGCCAGCAGCCGCGGTAACACGTAGGGTGCGAGCGTTGTCCGGAATTATTGGGCGTAAAG>OTU206TGGGGAATATTGCGCAATGGGCGGAAGCCTGACGCAGCGACGCCGCGTGGGGGATGACGGCCTTCGGGTTGTAAACCTCTTTCGCCAGGGACGAAGCTTTTTGTTGACGGTACCTGGAGAAGAAGCACCGGCCAACTACGTGCCAGCAGCCGCGGTAACACGTAGGGTGCGAGCGTTGTCCGGAATTATTGGGCGTAAAG>OTU228TGGGGAATATTGCGCAATGGGCGGAAGCCTGACGCAGCGACGCCGCGTGGGGGATGACGGCCTTCGGTTGTAACCTCTTTCGCCAGGGACGAAGCTTTTTGTGACGGTACCTGGAGAAGAAGCACCGGCCAACTACGTGCCAGCAGCCGCGGTAACACGTAGGGTGCGAGCGTTGTCCGGAATTATTGGGCGTAAAG
